# Cement beads and cement spacers: indications, techniques, and clinical results

**DOI:** 10.1097/OI9.0000000000000118

**Published:** 2021-06-15

**Authors:** Govind Shivram Kulkarni, Sunil Kulkarni, Sushrut Babhulkar

**Affiliations:** a GSK Orthopedic and Trauma Institute; b GSKs Fracture and Orthopedic Hospital, PG Institute of Swasthiyog Pratishthan, Miraj; c Sushrut Institute of Medical Sciences, Nagpur, India.

**Keywords:** antibiotic cement nails, antibiotic cement spacers, beads, infected nonunion, open fractures

## Abstract

**Objectives:**

To report our experience on the use of antibiotic coated nails (ACN) and cement beads for the management of bone infections.

**Materials and methods:**

Infected nonunion (INU) cases were classified as: Type I (mild infection with no gap), Type II (moderate with good alignment, severe infection, gap <3 cm, no deformity), Type III (severe infection with gap ≥3 cm, deformity and limb shortening). Treatment involved either the insertion of ACN and cast (Type I), insertion of ACN, beads and external fixator (Type II), or Ilizarov methodology (Type III). A subset of 28 open fractures were admitted with severe contamination or delayed presentation with established infection and treated with debridement, ACN insertion, and antibiotic beads placed in soft tissue dead space areas.

**Results:**

Results of 133 cases were classified excellent, good, and poor. Type I INU reported 40 excellent and 22 good results. Type II INU reported 28 (39%) excellent, 30 (43%) good, and 13 (18%) poor results. Poor results were due to uncontrolled infection and knee stiffness. Three patients required knee fusion and 1 required amputation. Fracture union was reported in 68 cases. Four of the 28 Gustilo grade III open fractures treated with ACN developed infected nonunion and had poor function caused by stiff knees.

**Conclusions:**

An antibiotic impregnated cement nail (ACN) fills the dead space and elutes high concentrations of antibiotics providing some mechanical stability. We recommend the adjunct use of an ACN for the management of INU cases and for use in select cases of Gustilo grade III open fractures.

## 1 Introduction

Development of postoperative infection after fracture fixation, infected nonunion (INU), and chronic osteomyelitis continues to be challenging to manage. Once biofilm is formed at the infection site, it is extremely difficult to eradicate the infection as the biofilm protects the bacteria (antibiotics do not penetrate the biofilm) thus contributing to the chronicity of infection.^[1,2,3]^ In our institution, the principles we follow to treat bone infections include: thorough surgical debridement with dead space management; stability of fracture/nonunion from treatment at day 1 through healing; administration of systemic and/or local antibiotics; soft tissue reconstruction; correction of any deformity (angular, rotational, length discrepancy), thus restoring the anatomy and facilitating optimum functional recovery.

Dead space management remains an essential step in the eradication of infection. The dead space created after debridement is usually filled with body fluid which could be a culture media for pathogens. Buchhloz^[4]^ was the first to use polymethyl methacrylate (PMMA) cement in the 1970s for the management of dead space. Since then, cement spacers have become the gold standard to treat dead space and the standard carrier for local antibiotic delivery for musculoskeletal infections. Interestingly, the use of local antibiotics does not increase the serum antibiotic concentration, and hence the risk for toxicity is considerably reduced.^[5]^

PMMA cement can be customized to different shapes and forms. Currently, PMMA cement spacers loaded with antibiotics are used as follows: in the treatment of joint infection after arthroplasty and in spine surgery as a space filler; in the 2-stage Masquelet technique for the control of infection and induction of the induced membrane; in open fractures as bead pouches (antibiotic beads placed in dead space areas in soft tissue); in the treatment of intramedullary sepsis (antibiotic impregnated cement nails [ACN]); and in the treatment of chronic osteomyelitis cases.

The main purpose of this study is to discuss the techniques and major indications for the use of cement spacers in our practice, and to present the clinical results of patients treated with ACN.

## 2 Indications for use of antibiotic coated nail and beads

The indication for ACN is intramedullary (IM) infection, a well-known complication of treating a fracture with IM nailing, plating, or external fixation. IM infection might also occur after conversion of an external fixator to IM nailing, limb lengthening over a nail, delayed presentation of open fracture with established infection, and hematogenous spread to the canal causing osteomyelitis. Part of the treatment process includes removal of the infected metal implant, reaming and irrigation of the medullary cavity, and the insertion of the ACN rod. The ACN is an ideal space filler offering some mechanical stability with good bony contact and high local release of antibiotics.

## 3 Preparation of ACN and cement beads

Pus from the infected area is sent for culture and sensitivity of organisms. Culture-specific antibiotics are used. A combination of vancomycin and gentamycin or tobramycin is used as a broad-spectrum antibiotic combination to treat both gram-negative and gram-positive organisms. We commonly use 2 to 3 g of vancomycin and 2 to 3 g of gentamycin or tobramycin. The antibiotic rod is prepared using PMMA cement (palacos 40 g) mixed with 4 to 6 g of antibiotic powder. When a soft dough is formed, it is wrapped around a 6 to 9 millimeter (mm) K-nail and is smoothened by rolling over a smooth surface such as a stainless-steel screw box. Herzog's bend is created for the tibial nail. While making the rod for the femur, a slight anterior curvature is made. Bumps on the nail, if any, are shaved off with a knife blade after the cement has hardened. Similarly, the diameter of the nail can also be reduced if necessary. The nail is kept in the air to help with polymer evaporation and to harden the cement. Leftover cement is then used for preparing beads.

Beads are threaded over 16-guage wire to prepare the chains and ends of the wire are bent to prevent beads from coming out of the chain. Beads should be round in shape and approximately 7 mm in size. The local concentration of antibiotics after the insertion of ACN and beads is substantially higher than that achieved with serum concentration.^[6]^ The local elution of antibiotics prevents bacterial growth and formation of biofilm, while it stabilizes the fracture nonunion and acts as a dead space filler.

## 4 Materials and methods

We conducted a retrospective review of patients treated in our institution from January 2008 to December 2019. The original technique described by Paley et al^[7]^ was modified by using a K-nail and manual rolling technique instead of using a thoracic tube and elastic nail. Overall, 178 (102 males) cases were eligible for review. A total of 98 tibia cases and 80 femur cases were reported, with a mean age of 48 years (range: 20–70 years).

Cases were classified as Type I (62 patients), Type II (71 patients treated by ACN), or Type III (45 patients treated by Ilizarov technique). A subset of 28 open fracture cases admitted with severe contamination or delayed presentation with established infection were also treated with debridement, ACN insertion, and beads in pouch technique. The patient subset included 6 Gustilo type I fractures, 12 Gustilo type II fractures, 6 Gustilo type III fractures, and 4 patients with infected nailing after primary surgery, which was performed elsewhere.

## 5 Authors classification of INU

The authors have developed a classification based on the severity of infection, apposition of fragments, and presence or absence of gap, shortening and deformity. Severity was judged by the quantity of pus drained, scarring of soft tissue, gap between fragments, and presence of callus. The classification indicates guidelines for treating INU. The INUs are classified into 3 types.

*Type I (mild):* Fragments are in opposition (no gap). There may be an implant in situ. On pressing around the wound, discharge of pus may be observed at the sinus or wound. Type I also includes a nondraining, dry wound for at least 3 months. Treatment is usually by a 1-stage surgical procedure with debridement, insertion of ACN, and plaster cast for tibial or hinged knee brace for femoral INU. Three-stage surgical procedures may be required to treat recurrent infection.

*Type II (moderate):* Fragments are in alignment with severe infection and no gap ≤3 centimeters (cm), shortening, or deformity. A small or large wound or sinus and a large amount of pus drainage will be present. An abscess deep down at the nonunion site may exist, which should be drained and debrided. An external fixator, preferably an Ilizarov ring fixator, gives additional stability to the nonunion after nailing, allowing compression or distraction and also deformity correction. If the wound is large, a plastic surgical reconstructive procedure may be needed to cover the wound. An active, nondraining wound with abscess and presence of fever is included in this type. Treatment is by a 3-stage procedure described below, and bone grafting may be necessary.

*Type III (severe):* Indicates severe infection with a gap >3 cm and deformity, shortening or combination of both. The Ilizarov method of treatment is required to correct the deformity and to treat the gap by bone transport. Forty-five patients classified as Type III were treated locally with antibiotic beads instead of ACN. Therefore, Type III is not discussed in this paper.

## 6 Three-stage procedure

### 6.1 Stage I surgery

The first stage consists of radical debridement, insertion of ACN, and stabilization by plaster cast or external fixator (monolateral or ring fixator). Steps include:

1. Thorough debridement: The first and single most important step in all 3 types of INU is thorough radical debridement. Debridement consists of excising the sinus tract plus all infected and necrotic or avascular tissues. Dead sclerotic bony ends are also removed. A 3.2 mm drill bit is passed at every centimeter from the end of each fragment, until blood oozes out from drill holes indicating the line of vascularity. The long axis of the bone is cut at a right angle by an oscillating saw. Punctate bleeding determined by the Paprica sign, should be seen at bony ends.
2. Reaming: The next important step of debridement is to ream the medullary canal to remove infected granulation tissue and small sequestrii. Frequent cooling by saline irrigation is important. A drill hole is created in the distal fragment to flush out the infected material which is collected for culture and sensitivity. Copious irrigation of the nonunion site and the medullary canal of both fragments by pulsed lavage with normal saline is performed.
3. Insertion of antibiotic intramedullary nail and beads, and wound care: An ACN with a 6 to 9 mm K-nail is inserted proximally into the distal fragment. The technique for inserting the ACN is the same as for antegrade interlocking nails. Dead space around the nonunion site is filled with antibiotic beads. If possible, the wound should be closed primarily. Open wounds with infection are treated with a vacuum-assisted closure (VAC) system after initial debridement. Healthy granulation tissue is evident in most cases after the first 3 or 4 VAC dressings. Large wounds require soft tissue reconstruction by muscle flap and/or skin grafting.
4. Stability: If stability is satisfactory after antibiotic rod insertion, Type I INU tibial fractures are treated with a full leg, knee in extension, plaster cast, which is inherently stable. ACN alone is not stable enough in Type II, especially to protect rotational stability, so additional external fixation is needed. A 2-ring Ilizarov construct or monolateral external fixator is used to further stabilize the ACN.
5. Insertion of pins and wires for Ilizarov frame: Metaphyseal areas of the femur and tibia are large, making it possible to insert Schantz pins and wires for llizarov frames 1 to 2 cm away from the path of the nail to prevent pin tract infection from spreading into the intramedullary canal. The distal tip of the nail stops 3 cm short of the ankle joint and the wires of the Ilizarov fixator are 1 cm away from the ankle joint. There is a clear space of 2 cm from the tip of the nail to the wires. Instead of an Ilizarov frame, monolateral external fixation may be used to stabilize the construct.

### 6.2 Stage II surgery

After the first surgery, patients are reassessed at 4 to 6 weeks. The status of infection is evaluated clinically, radiologically, and with laboratory tests (WBC, ESR, CRP). If exchange nailing with an interlocking IM nail is decided on, the external fixator is removed. A cast or brace is applied for 2 to 3 weeks for healing of the pin site wounds. In Type II INU cases, 15 patients had a persistent small gap and shingling, and bone grafting was carried out. In 5 patients, infection persisted with a draining sinus. These patients were treated with repeat debridement and a fresh ACN of a different antibiotic combination, such as tobramycin and ciprofloxacin.

### 6.3 Stage III surgery

If the infection is controlled or minimal, the wound is explored at 6 weeks. ACN beads are removed, the wound is debrided, and the canal is reamed. An IM interlocking nail is inserted and static locking is done. Cancellous bone grafting is performed if needed. If infection still persists, the wound is debrided and an ACN loaded with a different antibiotic is inserted.

### 6.4 Postoperative care

Patients are allowed to weight bear as tolerated as early as possible. Immediate joint mobilization is encouraged to avoid stiffness and contractures of soft tissue. Stage I lasts for a period of 4 to 6 weeks. Radiographs are taken every 4 weeks. If the fracture shows signs of healing, the same treatment of ACN with cast is continued. External fixation should be removed as early as possible to avoid restriction of joint movements, to prevent pin tract infection, and for the comfort of the patient.

## 7 Results

Case follow-up ranged from 1 to 24 years, with an average follow-up of 5 years and 7 months. Eight patients who could not travel to our institution participated in a telephone-based follow-up session with the help of a local orthopaedic surgeon. Results were classified into 3 groups: excellent, good, and poor. Excellent results indicate the fracture united, infection was controlled, and full function was regained. Good results indicate the nonunion was healed and infection was controlled, although patients had some restricted mobility of the knee and mild limp. Poor results indicate a stiff knee, persistent infection, nonunion, or amputation if required. Out of 133 cases reviewed, 72 cases involved the tibia and 61 the femur.

Sixty-two cases were classified as Type I. Forty cases united without reoperation and were rated as excellent. Twenty-two cases needed a second-stage procedure of exchange nailing with an IM interlocking nail and were rated as good. In 4 of the 22 cases, bone grafting from the iliac crest was required. Eight cases had restricted mobility of knee, but could flex the knee >90 degrees. Sixteen cases had a mild limp.

Of the 71 cases classified as Type II, 38 cases united and 15 cases required shingling and bone grafting. Eight cases had limb shortening of 3 to 4 cm. The patients were advised of limb lengthening over a slotted plate. Three patients agreed to limb lengthening and the procedure was successfully carried out. Five patients had a shoe raise. Twelve cases had a persistent or recurrent sinus for a duration of >1 year. Six patients healed, 6 had repeat debridement surgery; in 5 of those cases the sinus healed, and in 1 case, the sinus persisted. In 10 patients there was mild flexion deformity with flexion being 0 to 90° degrees or more. In 6 cases knee fusion was done because of a stiff painful knee (3 cases had septic arthritis of the knee). In 3 cases, infection could not be controlled and nonunion persisted. These patients were advised repeat debridement but refused further surgeries. One case developed severe infection and septicemia, and was treated with amputation. Thirty patients had some restriction of movements of the knee ranging from 5° to 90°. They were grouped as good results. Of the 71 Type II cases, 28 cases were rated as excellent, 30 cases were rated as good, and 13 cases were rated as poor. Cases and complications are summarized in Tables 1–3.

**Table 1:** Complications

Type	Recurrent infection NO (%)	Exchange nailing	Bone grafting NO (%)	Stiff knee NO (%)	Knee fusion	Failed to unite	Limb shortening
Type I (62 cases)	10 (16%)	22 (35%)	5 (8%)	6 (9.6%)	–	–	–
Type II (71 cases)	14 (20%)	6 (8%)	15 (21%)	6 (8%)	6 (8%)	3 (4%)	6 (8%)

**Table 2:** Results

Type	(No)	Excellent (No %)	Good (No %)	Poor (No %)
Type I	62	40 (64%)	22 (36%)	Nil
Type II	71	28 (39%)	30 (43%)	13 (18%)
Total	133	68 (51%)	52 (39%)	13 (10%)

**Table 3:** Results

Type of NF	No. of patients	Results			BG		Restricted knee movement	Limb shortening		Recurrent sinus		Amputation
		Excellent	Good	Poor		SPK		LL	SR	HB	PS	
Type I	62	40	22		4		8					
Type II	71	28	30	13	15	6	10	3	5	10	1	1

BG, bone graft; HB, healed after debridment; LL, limb lengthening; PS, persistent sinus; SR, shoe raise.

## 8 Use of ACN in open fractures

Indications for ACN and beads in open fractures of the tibia and femur were: wound was severely contaminated with mud and sand deeply embedded in the muscles and wound required multiple debridements and plastic surgery; late arrival of patient (after 4 days or weeks from initial injury with infection settled and with pus collection in the wound); late arrival of patient (1 or more months after infection after nailing or plating); farmyard injuries with high risk of infection.

Radical debridement is the most important step to improve outcomes. The ACN is inserted with the addition of an Ilizarov external fixator (our preference to monolateral fixator). Once the infection is under control, ACN and external fixator is removed and replaced with an interlocking IM nail or plate with an interval of 2 to 3 weeks allowing time for the pin tract infection to settle. Culture-specific antibiotics are added in the cement used to prepare ACN and beads. Antibiotics are given systemically for 3 to 4 weeks (1-week intravenously and 2–3 weeks orally). All 28 cases had additional external fixation (16 Ilizarov, 6 monolateral, and 6 plaster cast).

Six Gustilo grade I open fracture cases were reported with a puncture wound and delayed presentation of >4 days. Two united without second surgery, 4 had exchange interlocking IM nailing. All 6 united within 6 to 10 months.

Twelve Gustilo type II open fracture cases were reported. All had ACN and external fixation. External fixation was removed at 4 to 6 weeks and exchange interlocking IM nailing was done. Three cases required a third surgery, consisting of shingling and cancellous bone grafting from the iliac crest at 4 months after interlocking IM nailing.

Six cases of Gustilo open type III injuries presented 1 to 3 weeks after injury. All were treated elsewhere with nailing or plating and all had infection. Three cases required plastic surgery in the form of a local or distant flap and were treated with ACN and unilateral external fixation. The other 3 cases were treated with ACN and Ilizarov external fixation. After 4 to 6 weeks exchange interlocking IM nailing was performed. Two of these cases required bone grafting.

Four cases were closed fractures treated elsewhere with nailing and were admitted to our institute 1 to 3 months after the first surgery with frank infection. These patients were treated with debridement, ACN insertion, and external fixation. After 4 to 6 weeks, ACN was exchanged to an interlocking IM nail. Two needed bone grafting. Three cases united within 6 to 8 months, and 1 had nonunion which was treated with exchange nailing with an additional 8-hole 4.5 mm pate and bone grafting.

## 9 Discussion

In this study we found that results of using ACN for treating INU and a subset of open fractures were very good. Klemm and Seligson^[8,9]^ were the first to use a cement stick that did not provide any stability. Paley and Herzenberg^[7]^ were the first to use antibiotic impregnated cement rods using nails and thoracic tubes. Since then several other authors have reported their results using antibiotic impregnated nails.^[10,11,12,13,14,15]^

Intramedullary infection is a well-recognized complication of internal fixation by nailing or plating, with the infection spreading throughout the medullary canal. Multiple points or the entire canal may be involved in pin tract infections after external fixation.^[4]^ It is important to ream the canal to remove all infected granulation tissue. Reamer irrigation aspiration (RIA) may improve debridement of the canal.^[16,17]^

We have classified INU into 3 types based on the severity and gap. We found the classification helpful in our treatment strategy. Shyam et al^[14]^ have shown that a gap of >6 cms leads to a high failure rate. The authors do not recommend use of ACN for cases with a gap of >6 cm.^[13]^ In our experience, we think the critical gap is 3 cm which can be treated by ACN. Gaps >3 cm require other forms of treatment such as bone transport or the Masquelet technique. Jain classifies INU into 5 types and although this classification appears to be complicated, one can argue that it is rather comprehensive.^[18]^

In our opinion there are distinct advantages of using ACN and beads. A high local concentration of antibiotic is established, 200 times more than the serum concentration,^[1,7]^ which is adequate to kill even resistant organisms. ACN along with cast, brace, or external fixation provides adequate stability and also fills the dead space of the medullary canal. Noteworthy, the high concentration of antibiotics has minimal influence on new bone formation.^[6]^ Four grams of antibiotic in 40 g of cement will not cause any mechanical instability. It has been reported that the ratio of antibiotics to cement should not exceed 10% to have negligible effect on mechanical stability.^[19]^

We are aware that there is some controversy regarding the rate of antibiotic elution. It is biphasic with a high early elution rate followed by slow and sustained release as time progresses. Elution shows exponential decline after day 1 of implantation.^[3]^ At the end of the first week, 90% of the antibiotic has eluted. The remaining 10% of the antibiotic elutes over the next 6 to 8 weeks.^[3]^ Overall, clinically effective elution is up to 6 to 8 weeks, and as such, both the ACN and the beads can reduce the use of systemic antibiotics.^[20]^ Interestingly, hip spacers after infected arthroplasty show concentrations of antibiotic at 6 weeks; these same pharmacokinetics may be applied to antibiotic nails.^[21]^ The rate of elution depends on the type of cement, type and amount of antibiotic used, and how it is mixed. High porous cement elutes more antibiotic and for a longer period.^[6]^ Elution is improved with porosity of cement and increasing surface area.^[22]^ Adding another antibiotic not only increases the activity spectrum but also increases the antibiotic elution rate. Combinations of antibiotics have a synergistic effect. Elution also depends on the type of cement used with palacos cement showing the highest elution rate. This rate was explained by its inherent porosity.^[23]^ Finally, hand mixing the cement and antibiotic with a spatula has been shown to increase elution by increasing porosity of cement. Hand mixing also does not crush antibiotic crystals as can happen with device or vacuum mixing.^[24]^

After a period of 6 weeks when most of the elution has subsided, ACN acts as an inert implant. Therefore, ACN should be removed before 6 to 8 weeks and then an interlocking IM nail is inserted. After 8 weeks, if infection still persists, a fresh ACN with different culture-specific antibiotics is inserted. If the infection is mild or minimal, interlocking intramedullary nailing is used alone.

After a prolonged period of implantation, bacterial colonies are detected on the antibiotic cement and even have the potential ability to develop resistance to gentamycin despite preoperative susceptibility to local antibiotic.^[25]^ In Buchholz's classical work, he has shown antibiotics placed within bone cement did not necessarily correlate with culture-based sensitivity of organisms.^[26]^ Most bacteria, defined as antibiotic resistant when exposed to a very high concentration eluted by local antibiotics, become sensitive to the same antibiotic.^[21]^ On the other hand, even with high concentrations of antibiotics (up to 20%), colonization of the spacer or antibiotic rod can still occur.^[25]^

Klemm^[20]^ was the first to use antibiotic beads in cases of osteomyelitis. Cement beads fill the dead space and also allow high concentration of local antibiotics. Beads should be 7 mm in size to increase the surface area for better elution. Beads ideally should be removed at 3 to 6 weeks postsurgery.

Antibiotics commonly used for ACN are vancomycin, gentamycin, and tobramycin. Other antibiotics that have been used include cefazolin, ciprofloxacin, clindamycin, teicoplanin, erythromycin, colistin, and cefotaxime.^[27]^ Amphotericin B is used primarily for fungal infection.^[27]^ Systemic antibiotics are given intravenously for 7 days and then orally for 2 to 4 weeks depending on the severity of infection and the type of bacteria. The ideal antibiotic for ACN should fulfill some prerequisites: easily placed, removed, changed; high concentration locally; no allergic reaction; inexpensive; broad spectrum effective against both gram-positive and gram-negative cocci and multiresistant organisms; thermostable; available in powder form; no systemic toxicity; and satisfactory elution.^[1]^

Despite the advantages of ACN, one should also consider potential complications related to its usage. One of the common complications is cement debonding, as reported by Thonse.^[28,29]^ In our cohort of patients, we did not observe a single case of cement debonding after use of a 6 to7 mm K-nail, which was routinely used. Cement gets locked into the canal and around the nail due to its clover leaf configuration. In 1 series of 21 cases treated with ACN, there was nail breakage in 2 cases.^[14]^ We believe that this breakage was caused by not supporting the ACN by a plaster cast or external fixation, augmenting the fixation. ACN alone is not stable especially in regard to rotational stability. Stability of the construct is crucial for bone healing. Nonunions can unite even in the presence of infection if there is good stability of the construct.^[30,31]^ The worst scenario is a combination of active infection with instability, which perpetuates infection creating a vicious circle which ultimately leads to failure.

Overall, complications in this series were mostly related to the anatomical location around the knee (postoperative infection in distal femur fracture). Persistence of infection requiring repeated debridement was the cause of treatment failure.

This study had certain limitations. It is a retrospective study with possible bias for the selection of patients and the mode of treatment. Twenty-two patients were lost to follow-up. We have not compared our method of preparation of antibiotic rods with other methods. However, we think our method is simple and is not associated with any debonding of cement because of K-nail use. Strengths of this paper are the number of patients recruited and 90% good to excellent results. All cases were operated on under supervision of one surgeon (first author).

In conclusion, the use of ACN and beads in INU achieved good infection control. Complications, however, are to be expected in these challenging cases. Our classification of INU into 3 types according to the severity of infection and gap provides guidelines for treatment. Finally, the use of ACN and beads in a subset of open fractures, where infection has settled due to late presentation or farmyard injuries, appears to be a good option of treatment.
